# PReS-FINAL-2242: Familial Mediterranean Fever - first experiences in Slovakia

**DOI:** 10.1186/1546-0096-11-S2-P232

**Published:** 2013-12-05

**Authors:** T Dallos, L Lukáčiková Gálová, E Macejková, N Toplak, M Debeljak, L Kovács

**Affiliations:** 1 2nd Department of Paediatrics, Comenius University Medical School, Bratislava, Slovakia; 2 Department of Cardiology, University Hospital Nitra, Nitra, Slovakia; 3 Rheumatology outpatient office, Banská Bystrica, Slovakia; 4 Department of Allergology, Rheumatology and clinical Immunology, University Children's Hospital, University Medical Centre, Ljubljana, Slovenia; 5 Center for Medical Genetics, University Children's Hospital, University Medical Center, Ljubljana, Slovenia

## Introduction

Familial Mediterranean fever (FMF), resulting from mutations in the MEFV gene, is the most prevalent genetically determined autoinflammatory disease with the highest occurrence in the south-eastern Mediterranean's and Armenia. In central Europe, experience with FMF is limited, as in Slovakia, where no cases have been reported, so far.

## Objectives

To summarize the experiences with FMF in Slovakia.

## Methods

Paediatric rheumatologists were contacted and a search of local literature was carried out to identify and collect available retrospective clinical data of patients with FMF.

## Results

5 patients (1 male, 4 female, 2 related, 2 children and 3 adults) diagnosed with FMF based on the clinical picture and genetic analysis could be identified. Recurrent episodes of fever, abdominal and thoracic pain were present in all patients. Additional symptoms were arthralgia/arthritis (n = 3), splenomegaly (n = 1), hepatomegaly (n = 1), lymphadenopathy (n = 1) and exanthema (n = 1). In spite of significant diagnostic delay (4,5 - 30,0 years from onset of clinical symptoms), no patient has developed amyloidosis. Only well-known mutations (M694V, E148Q, V726A, E167D and F479L) were identified. All patients responded well to colchicine therapy (1 partial, 4 complete response) with good tolerance. Interestingly, only one patient originated from a high-risk population (Egypt), all other were of white Slovak native origin.

## Conclusion

FMF due to common MEFV mutations does occur in Slovak low-risk central European population. Due to its low prevalence and resulting lack of clinical expertize diagnosis may be significantly delayed, thus exposing patients to an increased risk of AA amyloidosis. Further undiagnosed cases are to be expected in our population.

## Disclosure of interest

None declared.

